# Is the asymmetry between the vertebral arteries related to cerebral dominance?

**DOI:** 10.3906/sag-1904-161

**Published:** 2019-12-16

**Authors:** Ahmet VURAL, Esin Derin ÇİÇEK

**Affiliations:** 1 Department of Radiology, University of Health Sciences, Fatih Sultan Mehmet Training and Research Hospital, İstanbul Turkey

**Keywords:** Cerebral dominance, vertebral arteries, handedness, Doppler ultrasonography

## Abstract

**Background/aim:**

The two vertebral arteries (VAs) are usually unequal in size; the left one is generally larger than the right one. It was hypothesized that the asymmetry results from the need of the dominant cerebral hemisphere for more glucose and oxygen, i.e. more blood supply. In this study, we aimed to test this hypothesis in patients by evaluating their arterial diameter and hand preference, as it is the most common criterion to determine the dominance of the hemisphere.

**Materials and methods:**

The study was performed with 844 participants who consented to participate in the study. We identified the dominant cerebral hemisphere by asking participants about their hand preference. Then we measured both the VA diameter and VA flow volume by Doppler ultrasonography. After demonstrating the asymmetry, correlation was tested.

**Results:**

Among 844 participants included in the study, the mean diameter of the right VA was 3.14 ± 0.35 mm and that of the left VA was 3.41 ± 0.54 mm, while the mean flow volume of the right VA was 119.21 ± 44.98 mL/min and that of the left VA was 151.45 ± 57.26 mL/min. It was recorded that 771 (86.43%) participants were right-handed and 73 (8.18%) were left-handed.

**Conclusion:**

No significant relationship was found between the increased blood demand of the dominant cerebral hemisphere and the vertebral artery dominance.

## 1. Introduction

The concept of hemispheric dominance in cerebral processes was first revealed by Broca in 1860 [1]. It could be thought that the dominant cerebral hemisphere may require more blood supply due to increased demand for oxygen and nutrients [2]. However, it is unclear whether the asymmetry in the cerebral blood flow is a determinant of the cerebral dominance [3].

In pre- and postmortem studies, the mean vertebral artery (VA) diameter was found to be larger on the left side than the right side [4,5]. Zaina et al. speculated that the vascular demands of the brain could lead to the asymmetry during the embryological formation [6]. Since hand preference is one of the indicators of cerebral hemisphere dominance, it could be expected that the left cerebral hemisphere will be dominant among right-handed people and vice versa. Based on this hypothesis, Cagnie et al. investigated the relationship between the VA diameters and hand preference in 50 subjects but could not prove a statistically significant correlation [7]. This may be due to the small number of subjects enrolled in the study. Therefore, we aimed to test this hypothesis with a larger group of participants (844 subjects). We analyzed arterial diameter and blood flow volume in correlation with right- or left-handedness. 

Additionally, to investigate the embryological origin differences as another hypothesis of diameters of vertebral arteries, diameters of the right VA with the right subclavian artery (SCA) and the left VA with the left SCA having similar origins were compared.

## 2. Materials and methods

The patients admitted to our clinic due to various reasons were asked if they wanted to participate in the study (n = 892). Patients with a history of significant neck pain, atherosclerosis, or cardiovascular disease were excluded. Hand preference was determined by ten questions in the Oldfield handedness questionnaire modified by Geschwind and Behan [8–10]. The required explanations about the importance of the questionnaire were made to ensure that the questions were answered correctly. Features required to be considered by the participants were specified. Through this survey, hand preference while performing some actions such as writing, painting, throwing a ball or stone, holding scissors, brushing teeth (hand holding the brush), holding a knife (cutting bread), holding a fork (without a knife), holding a hammer (when nailing), holding a matchstick while striking a match, and holding the cap while opening a bottle was examined. “Left hand”, “both hands”, and “right hand” responses were scored as –10, 0, and +10 points, respectively. The total ensuing score was evaluated according to the Geschwind score proposed by Tan. Geschwind scores range from –100 to +100. Negative scores show left-handedness, and positive scores show right-handedness. According to this scoring, those with scores between +40 and +100 were evaluated as right-handed, between –30 and +30 as ambidextrous, and between –100 and –40 as left-handed [11,12].

**Figure 1:**
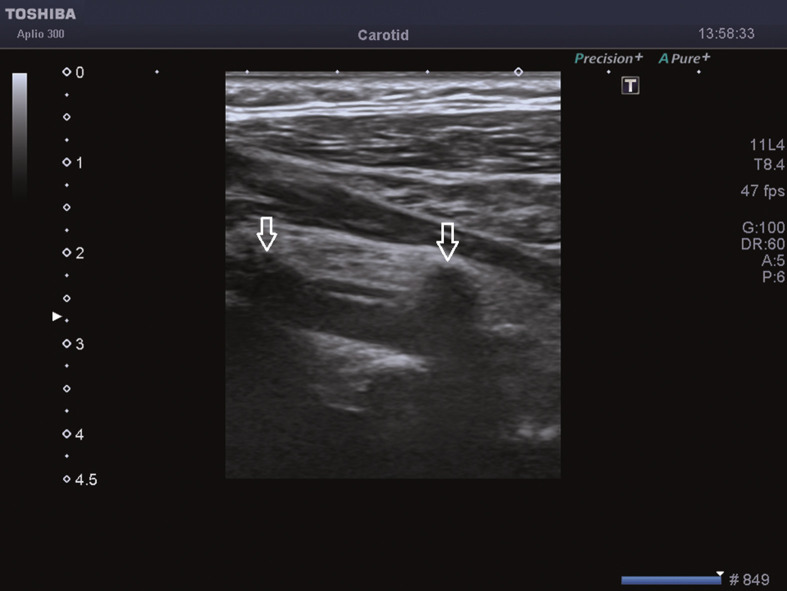
Normal vertebral artery of 38-year-old man. Gray-scale sonograms show vertebral artery below vertebral vein, both visualized between shadows from transverse processes of spine (arrows).

**Figure 2:**
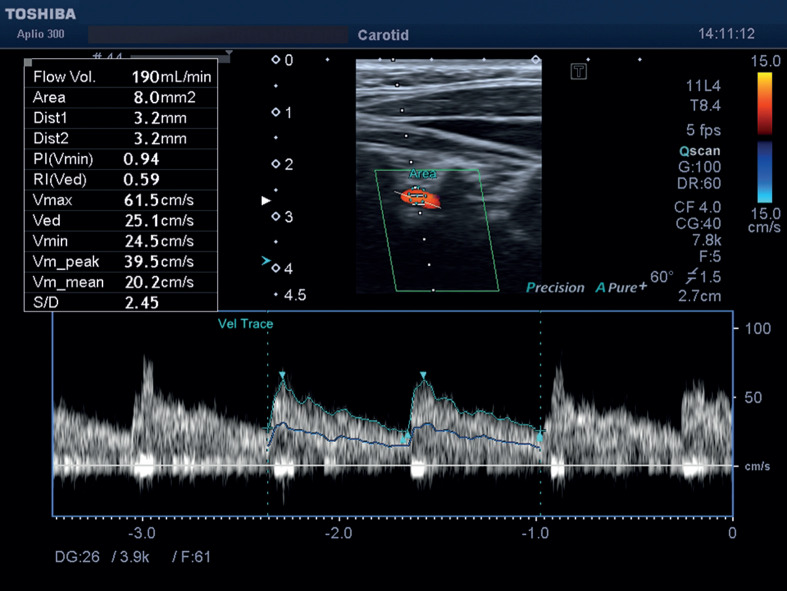
The left vertebral artery with normal flow parameters, flow volume, and diameter is observed with Doppler ultrasonography exam.

The examination of the vertebral arteries and subclavian arteries with duplex Doppler USG (Toshiba Aplio 300, Toshiba Medical Systems Corporation, USA) was performed by an experienced radiologist. All subjects were positioned supinely with the head in a neutral position. Both VA diameter and flow volume were measured with a 7.5-MHz linear probe between the fourth and fifth or fifth and sixth cervical vertebral transverse processes of both sides (Figure 1 and Figure 2). Similarly, in the same position, SCA diameters were measured at 1 cm distal from both sides of the VA in the supraclavicular area using the same probe. 

The study was approved (permit no: 13/2017) by the institutional research ethics committee for human clinical investigations and conformed to the protocols of the Declaration of Helsinki. Written informed consent was obtained from all patients enrolled in the study.

### 2.1. Statistical analysis

When evaluating the findings obtained in this study, IBM SPSS Statistics 22 (IBM Corp., Armonk, NY, USA) was used. The normal distribution of the parameters was evaluated by the Shapiro–Wilk test and the parameters were detected to have normal distribution. In the evaluation of the study data, besides descriptive statistical methods (mean, standard deviation, frequency), Student’s t-test was used in comparison of the quantitative data parameters of the two groups, and the paired samples t-test was used for intergroup comparisons. Pearson correlation analysis was used to examine the correlation between the parameters. Significance was evaluated at P < 0.05.

## 3. Results

In our study, 771 (86.4%) of the participants (n = 892) were right-handed, 73 (8.2%) were left-handed, and 48 (5.4%) were using both hands. The participants who used both hands were excluded from the study. The final evaluation was done for 844 cases (after excluding ambidextrous subjects). Two hundred and ninety (34.4%) were males and 554 (65.6%) were females. The mean age of the patients was 52.24 ± 12.64 years. The age range of the participants varied between 20 and 78. It was determined that 771 (91.4%) participants were right-handed and 73 (8.6%) were left-handed.

In right-handed subjects, the mean left VA diameter was statistically significantly larger than the right VA diameter (P: 0.000; P < 0.05).

In left-handed subjects, the mean left VA diameter was statistically significantly larger than the right VA diameter (P: 0.000; P < 0.05).

There was no statistically significant difference with regards to the right and left VA diameter between right-handedness and left-handedness (P > 0.05) (Table 1).

**Table 1:** Vertebral artery diameter (mm) and vertebral artery flow volume (mL/min) evaluation.

	Right Hand	Left Hand	^1^P
	mean ± SD	mean ± SD	
Right vertebral artery diameter	3.13 ± 0.34	3.18 ± 0.40	0.258
Left vertebral artery diameter	3.50 ± 0.55	3.45 ± 0.49	0.462
^2^P	0.000*	0.000*	
Right vertebral artery flow	118.55 ± 44.42	126.16 ± 50.41	0.167
Left vertebral artery flow	151.95 ± 57.12	146.16 ± 58.82	0.409
^2^P	0.001*	0.066	

^1^ Student t-test 2 ^2^ Paired samples t-test *P < 0.05

There was no statistically significant difference between the right and left VA flow volume averages for right dominant hands and left dominant hands (P > 0.05).

In right-handed subjects, the mean left VA flow volume was significantly higher than the mean right VA flow volume (P = 0.001).

In left-handed subjects, although the mean left VA flow was higher, there was no statistically significant difference between the right and left VA flow volume averages (P = 0.06). 

There was a statistically significant difference between the mean right VA diameter and the mean left VA diameter (P < 0.05). 

The difference between the mean right VA flow volume and the mean left VA flow volume was statistically significant (P < 0.05) (Table 2).

**Table 2:** Comparison of right and left vertebral artery diameter (mm) with vertebral artery flow volumes (mL/min).

	mean ± SD	^1^P
Right vertebral artery diameter	3.14 ± 0.35	0.000*
Left vertebral artery diameter	3.49 ± 0.54	
Right vertebral artery flow volume	119.21 ± 44.98	0.000*
Left vertebral artery flow volume	151.45 ± 57.26	

^1^Student t-test * P <0.05

The mean diameter of the right SCA was measured as 9.28 ± 0.48 mm, and the mean diameter of the left SCA was measured as 9.16 ± 0.53 mm.

There was no statistically significant correlation between the right VA diameter and the right SCA diameter (P > 0.05).

There was no statistically significant correlation between the left VA diameter and the left SCA diameter (P > 0.05) (Table 3).

**Table 3:** Correlation analysis between vertebral artery diameter and subclavian artery diameter.

	Vertebral artery diameter –Subclavian artery diameter	
	r	P
Right	0.067	0.053
Left	–0.028	0.410
Pearson correlations		

## 4. Discussion

In this study, we investigated whether right-handed people have a dominant left VA, or whether left-handed people have a dominant right VA.

It was previously stated that the individuals who used their left hand or both hands constituted approximately 2–30% of the human population [13]. Depending on the criteria used to determine the hand preference, there may be a difference of around 10%. Some studies reported that the use of the right hand increased with age [14]. In our study, it is reported that 86.43% of the participants were right-handed, 8.18% were left-handed, and 5.38% were ambidextrous. As a cultural habit, families in Turkey tend to discourage their children from using their left hands [15]. However, the effects of such environmental factors are unknown [16]. 

There are different methods to determine the dominant VA in the literature, but there is no consensus. Jeng et al. stated that there should be at least 0.3 mm diameter difference between vertebral arteries to confirm an asymmetry [17]. However, Smith and Bellon stated that a minimum of 30% difference should exist between the arteries [18]. In our study, when the difference of 0.3 mm in diameter (as accepted by Jeng et al.) was taken as the criterion for dominance, the diameter of the left VA was more dominant in 58% of the cases while the right vertebral diameter was dominant for 19%. In 23% of cases, no dominance between the right and left was detected. The percentages observed in this study are different than the ones reported in the literature, where the left VA was found dominant in 35.5–46.5% of individuals and the right VA dominant in 22.4–35.7% [16,19–22]. In our study, left VA dominance was more prominent with a prevalence of 58%.

We determined that the diameter of the left VA was significantly larger in individuals preferring both the right and left hand. The left VA flow volume was found significantly higher in the right-handed group. The left VA flow volume was also higher in the left-handed, but it was not statistically significant. In most of the studies in the literature, although it is stated that there is a difference in favor of the left between right and left vertebral arteries, neither of them showed a significant difference [6,7,16]. In our study, when the right and left vertebral arteries were compared in terms of the mean diameters and mean flow volumes, the dominance of the left vertebral artery was found to be statistically significant (Table 2). The large variability in the VA diameter reported in different studies in the literature could be a result of the variation in methods and protocols.

Different theories were proposed to explain the asymmetry in VA diameters. The theory of the brain’s vascular demand was suggested, but not adequately investigated. Orlandini et al. stated that the arteries on the left side of the circle of Willis are larger than the ones on the right side, and this situation is related to the normal dominance of the left cerebral hemisphere [23]. There is a common agreement that the left and right brain hemispheres differ in anatomy and function. Hand preference in the normal population is accepted as one of the criteria for predicting cerebral dominance. It is shown that left hemispheric dominance is more common in right-handed individuals, while right hemispheric dominance is more pronounced in left-handed ones [24]. It seems tempting to relate the dominant nature of the VA to the high blood flow volume required to meet the increased demand of cerebral hemispheres. However, our finding does not provide evidence to support this hypothesis (Table 1).

The second hypothesis of this study is the difference of embryological development. Although there is no reference to this in the literature, one could assume that the diameter and flow difference of the vertebral arteries may be due to the difference in the embryological development of the left and right vertebral arteries. On the right side, the SCA (normally the artery where the VA emerges) is sourced by the brachiocephalic trunk, and on the left, the SCA is sourced directly from the aorta. In this case, vertebral arteries are rooted from the left horn of the aortic sac on the left side embryologically, while on the right side, they are rooted from the right horn of the aortic sac embryologically [25–27]. This situation may explain the diameter and flow volume differences. In our study, we compared the diameters of the right VA and the right SCA with the same embryological origin. Although the arteries rooting from the same embryological origin are expected to show similar characteristics in terms of dominance, our findings have not confirmed this. In the majority of subjects with predominant right or left VA, the right SCA diameter was larger, but no statistically significant difference was detected between the subclavian arteries. No significant correlation between the VA diameters and SCA diameters was detected (Table 3).

The current results should be evaluated within the limitations of our study. First, the vertebral artery is not the sole source of blood supply to the cerebral hemisphere. A study with internal carotid arteries can provide further evidence. Although the diameter of the subclavian arteries were included in the study, additional investigations are required to obtain detailed information on embryological origin.

In conclusion, our aim was to investigate the dominance of the left vertebral artery that attracted our attention in clinical practice. The first thought was to test the hypothesis that the dominant cerebral hemisphere might need more blood supply. Additionally, we wanted to evaluate the relation of embryological development with this asymmetry. As a result, we observed prominent dominance for left vertebral artery. However, there was no significant correlation between arterial dominance and cerebral hemisphere dominance. In addition, no correlation was found based on the evaluation of the diameters of arteries of the same embryological origin.
